# The effect of bioequivalent radiation dose on survival of patients with limited-stage small-cell lung cancer

**DOI:** 10.1186/1748-717X-6-50

**Published:** 2011-05-19

**Authors:** Bing Xia, Gui-Yuan Chen, Xu-Wei Cai, Jian-Dong Zhao, Huan-Jun Yang, Min Fan, Kuai-Le Zhao, Xiao-Long Fu

**Affiliations:** 1 Department of Radiation Oncology, Fudan University Shanghai Cancer Centre; Department of Oncology, Shanghai Medical College, Fudan University, Shanghai, China

## Abstract

**Background:**

To investigate the biological radiation dose-response for patients of limited-stage small-cell lung cancer (LS-SCLC) treated with high radiation dose.

**Methods:**

Two hundred and five patients of LS-SCLC treated with sequential chemotherapy and thoracic radiotherapy with involved-field between 1997 and 2006 were reviewed retrospectively. Biologically effective dose (BED) was calculated for dose homogenization and was corrected with the factor of overall radiation time. Patients were divided into low BED group (n = 70) and high BED group (n = 135) with a cut-off of BED 57 Gy (equivalent to 60 Gy in 30 fractions over 40 days). Outcomes of the two groups were compared.

**Results:**

Median follow-up was 20.7 months for all analyzable patients and 50.8 months for surviving patients. Considering all patients, median survival was 22.9 months (95% confidence interval, 20.6-25.2 months); 2- and 5-year survival rates were 47.2% and 22.3%, respectively. Patients in high BED group had a significantly better local control (*p *= 0.024), progression-free survival (*p *= 0.006) and overall survival (*p *= 0.005), with a trend toward improved distant-metastasis free survival (*p *= 0.196). Multivariable Cox regression demonstrated that age (*p *= 0.003), KPS (*p *= 0.009), weight loss (*p *= 0.023), and BED (*p *= 0.004) were significant predictors of overall survival.

**Conclusions:**

Our data showed that a high BED was significantly associated with favourable outcomes in the Chinese LS-SCLC population, indicating that a positive BED-response relationship still existed even in a relatively high radiation dose range.

## Background

Although concurrent thoracic radiotherapy (TRT) combined with chemotherapy represents the standard of care in the management of limited-stage small-cell lung cancer (LS-SCLC), the optimal radiation schedule and total dose for LS-SCLC remain topics of continuous debate [1,2]. In the landmark study of Intergroup Trial 0096 [3], Turrisi et al. demonstrated that twice-daily TRT of 45 Gy over 3 weeks yielded both superior local control (LC) and overall survival (OS) rate compared to once-daily TRT of 45 Gy over 5 weeks, strongly suggestive of enhanced dose intensification may improve LC which resulted in prolonged OS in LS-SCLC. Nevertheless, high frequency of local failure rate (36%) despite bid TRT [3] has led to investigations of higher doses of TRT. Higher dose up to 70 Gy of once-daily TRT for LS-SCLC is feasible, as have been showed in several retrospective and prospective small studies [4-7]. Also, the regimen of 61.2 Gy concomitant boost TRT was investigated in phase I and II studies by the Radiation Therapy Oncology Group (RTOG) [8,9]. However, none of these high dose regimens appeared to be superior to 45 Gy over 3 weeks in terms of tumor control rate even though tolerability were generally reported.

Multiple studies have confirmed that there is a radiation dose-response for SCLC but the radiation dose evaluated was often in the lower range of 25-50 Gy [10-12]. Choi et al. reported a positive dose-response relationship with a LC rate of 16%, 51%, 63%, and 78% for a radiation dose of 30, 40, 50, and 57 Gy (range 50-72), respectively [5,11]. But there was no significant difference in outcomes between patients treated with a median dose of 54 Gy (range 50-54) and those treated with a median dose of 63 Gy (range 55-72) in a subgroup analysis.

As SCLC presents the biological characteristics of sensitivity to treatment and early spread to distant sites, we really do not know whether further increase of TRT dose is necessary for LS-SCLC. Our concern is whether a dose-response relationship still exists for improved LC and OS in LS-SCLC when a certain threshold of TRT intensity has been reached. Unfortunately, few studies have been specifically addressed this critical issue for LS-SCLC. In order to evaluate if there is a dose-response relationship, the outcome of LS-SCLC patients treated consecutively at our centre with combination of chemotherapy and TRT with doses greater than 50 Gy were reviewed. Since radiation dose confounds both fractionation and overall radiation time (ORT), the biologically effective dose (BED) with ORT will be a more appropriate representative of the biological effect than the single physical dose. Thus we investigated the underlying BED-response relationship for LS-SCLC in this study.

## Methods

### Patients

Medical and RT records of all patients with LS-SCLC between 1997 and 2006 were reviewed. Patients were selected based on the initial diagnosis of LS-SCLC where definitive TRT with doses equal or greater than 50 Gy was carried out as a part of their treatment for this disease. All patients had histology confirmed SCLC by bronchoscopic, transthoracic biopsy or sputum cytology no less than twice. Pre-treatment staging procedures consistently included clinical history, physical examination, biochemical test, computed tomography (CT) scan of the thorax and abdomen, magnetic resonance imaging or CT scan of the brain, and bone scan. Limited-stage disease was defined as disease confined to one hemithorax which can be safely encompassed within a tolerable radiation field. Presence of an ipsilateral pleural effusion was classified as limited-stage if cytology was negative or if the effusion was small.

A total of 234 patients were identified as LS-SCLC in the period, 29 were excluded because they had undergone surgery (n = 14) or had been treated to dose < 50 Gy (n = 15). For all 205 patients with definitive chemoradiotherapy, median age at diagnosis was 62 years (range 35-83) and median KPS was 80 (range 60-100).

### Treatment Decision

Treatment strategies were determined on the basis of tumor status, patient's performance and comorbidities at the discretion of the treating oncologist, and referring to the clinical practice guidelines formulated in our centre. The majority of patients were given modified chemoradiotherapy because of concerns of serious toxicity from concurrent chemoradiotherapy and insufficient supportive treatment in developing country [13]. Generally, 2-4 cycles of induction chemotherapy were administered, followed by initiation of TRT within 1 week after the start of the last cycle of induction chemotherapy, and then 2-4 cycles of adjuvant chemotherapy delivered within a week at the end of TRT. Chemotherapy was a combination of platinum and etoposide regimen, typically delivered every 3-4 weeks per cycle. After the completion of TRT and chemotherapy, patients with a complete clinical/radiological response received prophylactic cranial irradiation (PCI) with 25 Gy in 10 fractions over 2 weeks. However, due to the poor treatment adherence to preventive intervention, only 12% of the patients undertook PCI in our study population.

### Thoracic Radiotherapy

During the period, TRT was delivered with megavoltage equipment (6-15 MV), and either two-dimensional or three-dimensional techniques were allowed. The gross target volume (GTV) was based on the restaging chest CT obtained after the last induction chemotherapy, including the primary tumor (post-chemotherapy) and all clinical/radiological involved lymphatic regions with a short-axis diameter ≥ 1 cm (pre- or post-chemotherapy). Elective treatment of clinically uninvolved lymphatic regions was not carried out. No specific clinical target volume (CTV) was used in this population. A margin of 1.0-1.5 cm was placed to form planning target volume (PTV) according the site and motion of the target (the margin of 1.5 cm was commonly used in the most of patients). Typically, patients with two-dimensional planning were treated with equally weighted AP-PA fields to 40-42 Gy, then boost by parallel opposed off cord oblique fields to the prescribed dose. For patients with three-dimensional planning, three to six coplanar photon fields were used and the prescribed dose was corrected for lung inhomogeneity. As for the dose fractionation scheme, both once-daily and twice-daily fractions were used in the period, which was chosen mainly depended on the attending physician's judgment and preference. For patients with once-daily TRT, a total dose of 50-70 Gy was administered at 1.8-2.5 Gy per fraction. For patients with twice-daily TRT, a total dose of 56 Gy at 1.4 Gy per fraction was delivered at intervals longer than 6 h, in 40 fractions over 4 weeks, which has been described previously [13].

### Radiation Dose Homogenization

To enable comparison of the physical dose values with different fractionation schemes, we calculated the BED using the linear quadratic formula that included the factor of ORT which could take into account for the accelerated proliferation during irradiation course [14].

Where n is the number of fractions, d is the fraction size, α/β ratio is 10 Gy, α is 0.3 Gy, T is the ORT considering that the first fraction was given on day 1, T_k _is the delay in proliferation in tumors ('kick-off time' is assumed to 21 days), T_pot _is the potential doubling time of the tumor clonogenic cells which is set to 3 days for SCLC [15]. In our study, patients received twice-daily TRT have intervals longer than 6 h between fractions, so the impact of incomplete repair to the BED was considered to be little and was not included in the formula.

### Treatment Toxicity

In this study, toxicity associated with TRT was reported as days of interruption during the course of TRT except for holidays and mechanical failures. The hematologic criteria for interruption included absolute neutrophil count ≤ 1000/mm^3^, neutropenic fever or sepsis, and platelet count ≤ 50,000 mm^3^. Loco-regional symptoms included severe esophagitis (i.e., severe dysphagia, intolerable pain, requiring IV fluids or tube feedings), and severe pneumonitis (i.e., severe coughing, dyspnea requiring oxygen inhalation, need to exclude tumor-related symptoms).

### Follow-up and Statistical Analysis

Generally, patients were followed up every 3-4 months for 2 years, then every 6 months thereafter. The survival status of patients lost to follow-up was updated with the information from the R.P.C Social Security System. OS was the primary endpoint of this study which was measured from the start date of any treatment to patients' death from any cause or the last follow-up. Only the first treatment failure was taken into account. Progression-free survival (PFS) was defined as the duration of survival without loco-regional recurrence or distant metastases. Local recurrence was defined as disease progression within the irradiated field alone or together with distant metastases (diagnosed within one month after the initial finding of failure), while progression of tumor out-of-field was not included in this analysis, provided that this kind of loco-regional recurrence can't be controlled by TRT intensification. Distant-metastases free survival (DMFS) was defined as the interval from the day that treatment initiated to the day of distant metastases occurred or the last follow-up. All endpoints were estimated by Kaplan-Meier model.

The Duke's experiences showed that LS-SCLC patients treated with approximately 60 Gy once-daily TRT have promising outcomes [4], and this dose is also the lower limit of 60-70 Gy in conventional fraction recommended by National Comprehensive Cancer Network (NCCN) [16]. Therefore, we divided patients into two groups with a cut-off of BED 57 Gy (equivalent to 60 Gy in 30 fractions over 40 days), with the hypothesis that high BED is associated with better outcomes. The OS, PFS, LC and DMFS between the two groups were compared using the log-rank test. Cox's proportional hazards model was used for multivariate analysis to estimate the simultaneous impact of factors on OS. All *p *values were two-sided, with *p *≤ 0.05 considered significant.

## Results

At the present analysis, 42 patients (20.5%) were alive, 153 dead (74.6%) and 10 censored (4.9%). Median follow-up time was 20.7 months (range 3.6-102.8 months) for all analyzable patients and 50.8 months (range 27.3-102.8 months) for patients alive. Considering all patients, median OS was 22.9 months (95% confidence interval [CI], 20.6-25.2 months); 2- and 5-year OS were 47.2% and 22.3%, respectively.

Of the 205 patients, 70 received BED ≤ 57 Gy (low BED group) and 135 > 57 Gy (high BED group). Table 1 provided a comparison of patient- and treatment-related factors between the two groups. No statistically significant imbalance was found in these variables except for the daily fractions. Twice-daily TRT was significantly more frequent in high BED group (*p *= 0.000). Additionally, it should be mentioned that we also evaluated the size of equivalent square field at anterior-posterior axis as an alternative indicator of tumor volume for each patient, considered that the prescribed TRT dose may be affected by the tumor volume. In some cases treated with three-dimensional conformal TRT, a virtual field was utilized to generate the size. As a result, there was no significant difference between the two groups.

**Table 1:** Patient and treatment characteristics

Characteristic	Lower BED Group (≤57 Gy)	Higher BED Group (>57 Gy)	*P*
Patients(n)	70	135	
Age(years)			
Median	62	60	0.574
Range	38-83	35-81	
Gender			0.713
Male	57(81.4)*	107(79.3)	
Female	13(18.6)	28(20.7)	
KPS			
Median	80	80	0.163
Range	60-100	60-100	
Weight loss>5%			0.178
Yes	21(30.0)	29(21.5)	
No	49(70.0)	106(78.5)	
LDH			0.517
≤220 IU/L	35(43.1)	34(35.3)	
>220 IU/L	25(26.4)	78(22.6)	
Unknown	10(30.5)	23(42.1)	
ISN			0.190
Yes	8(11.4)	25(18.5)	
No	62(88.6)	110(81.5)	
CHT cycles			0.620
Median	4	6	
Range	3-8	3-7	
SER			0.951
Median	81	72	
Range	34-262	30-236	
BED(Gy)			0.000
Median	53.6	58.5	
Range	41.3-56.9	57.1-66.1	
TRT fractions			0.000
Once-daily	52(74.3)	23(17.0)	
Twice-daily	18(25.7)	112(83.0)	
TRT technique			0.418
2D	52(74.3)	107(79.3)	
3D	18(25.7)	28(20.7)	
Size of TRT field(cm^3^)^#^			0.722
Median	132	137	
Range	54-210	46-228	
PCI			0.199
Yes	11(15.7)	13(9.6)	
No	59(84.3)	122(90.4)	

* Data in parentheses are percentages.

^# ^The size of equivalent square field at anterior-posterior axis

Abbreviations: BED = biologically effective dose with time correction; KPS = Karnofsky performance score; LDH = lactate dehydrogenase; ISN = ipsilateral supraclavicular nodes; CHT = chemotherapy; SER = the time from the start of any treatment to the end of chest irradiation; TRT = thoracic radiation therapy; 2D = 2 dimension; 3D = 3 dimension; PCI = prophylactic cranial irradiation.

**Figure 1:**
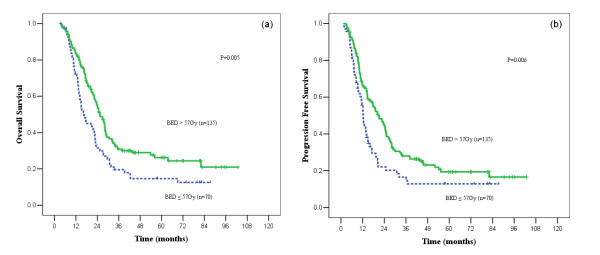
**Curves for overall survival (a) and progression-free survival (b)**. Comparison between biologically effective dose (BED) > 57 Gy and BED ≤ 57 Gy groups for patients with limited-stage small-cell lung cancer, both favouring the BED > 57G group.

The median OS for patients treated with low BED and those with high BED were 16.4 months (95% CI, 10.9-21.9 months) and 25.4 months (95%CI, 21.9-29.0 months); 2- and 5-year OS were 31.5% and 14.6%, 55.2% and 26.2%, respectively (*p *= 0.005, Figure 1a). The probability of PFS was significantly higher in high BED group than in low BED group (*p *= 0.006, Figure 1b).

**Figure 2:**
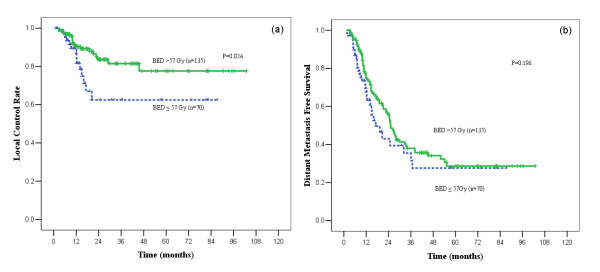
**Curves for local tumor control and distant-metastasis-free survival**. Comparison between biologically effective dose (BED) > 57 Gy and BED ≤ 57 Gy groups for patients with limited-stage small-cell lung cancer. (a) Patients in BED > 57 Gy group had significantly better local tumor control. (b) A trend toward better distant-metastasis-free survival was also found for the BED > 57 Gy group.

The sites of first relapse were recorded for 141 patients (68.8%). Table 2 listed the patterns of the first failure. In low and high BED group, local recurrence occurred as the first failure in 14 and 18 patients, respectively. The 1- and 2-years LC rates were 81.6% and 62.5% in low BED group, while 90.4% and 83.7% in high BED group, favouring the high BED group (*p *= 0.024, Figure 2a). The most common sites of distant metastasis were brain, bone, and liver. No statistically significant difference was found in DMFS between the two groups. However, a trend toward improved DMFS was noted in those patients receiving high BED (*p *= 0.196, Figure 2b).

**Table 2:** Patterns of first treatment failure

Treatment	No. of Patients	Distant Metastases Alone	Local-regional Recurrence		Censored Observations
				
			Alone or with Distant Metastases	Alone	
Lower BED group(≤57Gy)	70	28(40)*^b^*	21(30)	17(24)	21(30)
Higher BED group(>57 Gy)	135	60(44)	32(24)	20(15)	43(32)
All patients	205	88(43)	53(26)	37(18)	64(31)

Censored observations indicate patients without recurrences or loss of follow-up.

Data presented as the number of patients, with the percentage in parentheses.

Abbreviations: BED = biologically effective dose with time correction.

The most common acute complication was radiation esophagitis. There was no significant difference between the low and high BED groups in the incidence of Grade 3 esophagitis, defined as an inability to swallow solids, requiring narcotic analgesics or the use of a feeding tube (8.6% vs. 10.4%, *p *= 0.681). Seven patients (3.4%) experienced Grade 3 acute pneumonitis, defined as severe coughing or dyspnea requiring oxygen inhalation. There was no difference between the two groups in the incidence of Grade 3 pneumonitis (2.9% vs. 3.7%, *p *= 0.752). A total of 46 patients (22.4%) required treatment interruptions during TRT due to hematologic and/or loco-regional toxicities. The factors directly leading to treatment interruptions were esophagitis (40.4%), neutropenia (29.8%), pneumonitis (12.8%), nausea and vomiting, dehydration, and others (17.0%). The median duration of treatment "break" was 6 days (range 1-18). Thirteen patients (18.6%) in low BED group experienced treatment breaks, while 33 (24.4%) in high BED group did. No statistically significant difference was found in the incidence of interruptions as a function of BED (*p *= 0.339).

The effects of patient- and treatment-characteristics on OS are shown in Table 3. Univariate analysis showed that age ≤ 65 years, high KPS, weight loss ≤ 5%, high BED and PCI were significantly associated with improved OS. BED was also significantly associated with OS when analyzed as a continuous variable (*p *= 0.019). The time from the start of any treatment to the end of the TRT (SER) was not a significant factor for OS when SER was analyzed as a continuous variable (*p *= 0.530) or as a categorical variable based on the median value (*p *= 0.623). There were no significant differences in OS based on sex, lactate dehydrogenase, ipsilateral supraclavicular nodes, daily fraction, TRT technique, chemotherapy cycles or ORT.

**Table 3:** Univariate Cox regression analysis for overall survival

Factor	Hazard ratio	*p*	95% CI
Age (>65 y vs. ≤65 y)	1.667	0.007	1.147-2.424
Gender (female vs. male)	0.625	0.062	0.381-1.023
KPS (>80 vs. ≤80)	0.496	0.005	0.304-0.810
Weight loss (>5% vs. ≤5%)	1.820	0.004	1.205-2.749
LDH(>220 IU/L vs. ≤220 IU/L)	1.226	0.328	0.815-1.843
ISN (pre-treatment, yes vs. no)	1.408	0.166	0.867-2.287
Daily Fractions (once vs. twice)	1.112	0.575	0.768-1.609
TRT technique (3D vs. 2D)	0.821	0.357	0.539-1.249
CHT cycles (>5 vs. ≤5)	0.721	0.126	0.475-1.096
SER (>79 vs. ≤79 day)	1.097	0.623	0.758-1.587
ORT (>31 vs. ≤31 day)	1.356	0.106	0.937-1.964
BED (>57 vs. ≤57 Gy)	0.600	0.005	0.414-0.870
PCI (yes vs. no)	0.506	0.018	0.288-0.888

Abbreviations as in Table 1

**Figure 3:**
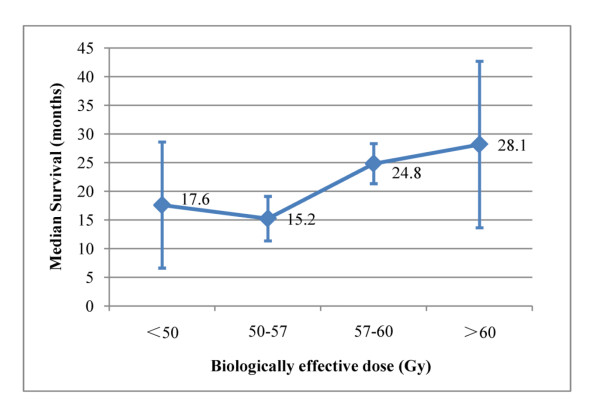
**Median survival as a function of biologically effective dose for limited-stage small-cell lung cancer**.

Multivariate analysis demonstrated that age ≤ 65 years, high KPS, weight loss ≤ 5% and high BED remained significantly correlated with improved OS (Table 4), while PCI was borderline associated with OS (*p *= 0.057). Figure 3 showed the median OS as a function of BED, a positive correlation was found although the slope of the BED-response seems relatively flat in the low BED region (*p *= 0.012).

**Table 4:** Multivariate Cox regression analysis for overall survival

Factor	Hazard ratio	*p*	95% CI
Age (>65 y vs. ≤65 y)	1.776	0.003	1.209-2.609
KPS (>80 vs. ≤80)	0.487	0.009	0.284-0.835
Weight loss (>5% vs. ≤5%)	1.693	0.023	1.076-2.665
BED (>57 vs. ≤57 Gy)	0.574	0.004	0.395-0.836
PCI (yes vs. no)	0.575	0.057	0.325-1.016

Abbreviations as in Table 1

## Discussion

This retrospective study showed that patients treated with BED > 57 Gy had significantly better LC, PFS and OS in LS-SCLC, indicating that patients could achieve benefits from high BED. This result is consistent with previous findings that TRT dose intensification improved LC, resulting in better outcomes in LS-SCLC [3,5,17]. Specifically, all patients in our study received TRT dose ≥ 50 Gy, which is high compared to doses adopted by previous studies [10-12]. Our results supported the hypothesis that a biologically dose-response relationship still existed even in a relatively high radiation dose range for LS-SCLC.

The radiation dose ≥ 50 Gy determined as the inclusion criteria for this retrospective analysis was based on our assumption that 50 Gy might be a conservative radiation dose for our LS-SCLC population with curative intent when sequential chemoradiotherapy was given, according to our previous study [13]. In the current analysis with a large sample size, patients who received BED > 57 Gy had significantly better LC rate, with a trend toward better DMFS. It was suggestive that further improving LC of the primary tumor with high BED may play a major role in reducing the risk of subsequent metastasis and that combination of improved LC and decreased distant metastasis would finally contribute to better OS in patients treated with high BED. In addition, our results showed that high BED was significantly associated with improved OS in patients with LS-SCLC, which is comparable to the findings of Schild et al., of which a strong positive correlation between BED and 5-year OS was shown with a reported Pearson correlation coefficient of 0.81 based on randomized trials that included various TRT programs for LS-SCLC [18]. Results from these studies suggested that for LS-SCLC, high BED which integrated the factors of TRT dose and ORT is important to achieve a better outcome.

Accelerated proliferation of tumor clonogens during radiotherapy has been shown to affect outcomes in many malignant solid tumors [19-22]. Two studies aimed to evaluate the impact of ORT on the results of TRT for non-small cell lung cancer showed that prolonged ORT accompanied with accelerated proliferation, was a major cause of treatment failure, which provided evidence that dose and time factors should be considered together for a reliable evaluation of a radiotherapy regimen [21,22]. Although the evidence for SCLC is not as strong for other solid tumors, it is believed that accelerated proliferation during TRT also exist in SCLC due to its characteristics of rapid doubling time and high growth fraction. Also, several studies explored the duration of radiotherapy indirectly indicated that extended ORT had a potential negative effect in the treatment of LS-SCLC [23-25]. Therefore, we think that it is more appropriate to include ORT for the examination of the relationship between BED and treatment outcomes in our analysis.

Indeed the biological radiation dose (without time correction) in the high once-daily [6,7] or the concomitant boost TRT [8,9] is higher compared to that used in the Intergroup Trial 0096 [3], but at the expense of prolonged ORT which could potentially lead to repopulation. This should be considered as one of the reasons for the less satisfying results in the several phase II clinical trials exploring high radiation dose [6-9], and 45 Gy twice-daily TRT should be considered as the standard treatment in LS-SCLC at this time. Currently, two ongoing randomized Phase III trials (CONVERT and CALGB 30610/RTOG 0538) are investigating the optimal dose of radiation in LS-SCLC [26,27]. The former uses a conventional regimen of 66 Gy in 33 treatments given daily as the experiment arm, and the latter includes two experiment arms: 70 Gy in 35 treatments given daily and 61.2 Gy in 34 treatments given daily, 5 days/week for 16 days, and then twice-daily, 5 days a week for 9 days. Both trials are using the 45 Gy twice-daily dose as the control arm, which will provide more data on the repopulation issue in LS-SCLC.

The distributions of patient- and treatment-related characteristics were similar for low and high BED groups except for daily fraction scheme. Twice-daily scheme was more frequent in high BED group than in low BED group (83% vs. 26%, *p *= 0.000). However, there was no significant difference in 5-years OS between the once-daily and twice-daily groups, (21.5% vs. 24.4%, *p *= 0.575). This indicated that high BED administered in once-daily scheme might lead to non-inferior outcomes compared with twice-daily scheme for LS-SCLC. Therefore, we believed that the difference of daily fraction scheme between the two groups had no apparent impact on our conclusions.

The differences between the physical constitution and patient compliance of the Asian and Western population may have resulted in different management of the LS-SCLC patients here in China. The Turrisi et al. schedule [3] had been tried in our centre, but unsuccessful predominantly because of severe esophagitis and bone marrow suppression occurred as a side effect in a large percentage of patients [13]. In the past, there was no sufficient nutrition support and granulocyte colony-stimulating factor supply, and sequential treatment of LS-SCLC was a more favourable treatment option in china, which was also common in other developing countries [28]. During the period, most physicians in our centre chose late commencement of TRT due to expected smaller treatment fields after initial shrinkage of the tumor mass occurring after induction chemotherapy. As more evidence supporting early administration of TRT emerging in recent years [29,30], more and more patients received early TRT in our centre.

The median OS were 22.9 months and 5-year OS was 22.3% in this study, which was within the range of other reports using early concurrent chemoradiotherapy [3,23]. The results were acceptable, although concurrent chemoradiothrapy was not used in this population and most of the patients were administered with TRT late. This might be explained by the facts that: 1) only those patients who completed induction chemotherapy and TRT doses ≥ 50 Gy were included in this analysis, this criteria might excluded some patients with poor prognosis and those who had poor compliance to treatment; 2) all the patients in this study received high dose TRT, to some extent, contributed to the improvement of the treatment outcomes.

Because it was difficult to accurately evaluate treatment toxicities in this retrospective study, interruption during TRT was used as an alternative indicator. The interruption occurred in 22.4% of the patients even when TRT was delivered with relatively high dose, which was similar to previous report [31]. The possible explanation for lower incidence of acute toxicity was that we used a modified schedule of chemoradiotherapy and TRT with involved-field irradiation technique for LS-SCLC, both of which were considered to possibly reduce the incidence of treatment toxicities. Nowadays, there have been many advances which contributed to making TRT dose intensification feasible, including imaging techniques, radiation planning and radiation delivery. Furthermore, there is a trend towards smaller fields with the omission of elective nodal irradiation [32], which will further help TRT intensification by limiting dose-dependently aggravated toxicity in radiotherapy. Nevertheless, the available data about treatment associated toxicities for LS-SCLC were generally based on old irradiation techniques with a large portal, which to a certain extent, limited the possible benefits from intensified TRT. Future studies should examine the beneficial and detrimental effects of high BED with modern irradiation techniques and an appropriate TRT portal.

This study had some limitations. Because only the site of first failure was recorded, data on local recurrence after distant metastasis were censored. Thus, it had the risk of obscuring the true LC rate. The issue of LC was further complicated by the difficulty in defining local failure. It was very hard to evaluate local failure accurately because of limited ability of imaging modality to discriminate the radiographic abnormality, which was also the reason for choosing OS as the primary end point in our study. We believed that OS could be more appropriate to evaluate the impact of TRT intensification on treatment outcomes than LC [33]. In addition, uncontrolled chemotherapy dose in this population could be another potential confounder. It is well known that chemotherapy is the corner stone for the management of SCLC, thus insufficient chemotherapy dose may offset the possible benefits from escalation of BED, leading to compromised treatment outcomes. In this study the relatively high incidence of distant metastasis might be due to inadequate chemotherapy intensity. Therefore, it was believed that the benefits from escalation of BED would become more prominent when sufficient chemotherapy dose intensity was given in a prospective study. Lastly, the majority of patients in our report received modified chemoradiotherapy rather than a standard regimen of concurrent chemoradiotherapy, which seemed to be a possible confounder. While this work is intended to investigate the relationship between radiation dose and treatment outcomes, we considered that the factor of timing and sequencing of TRT has little influence to our conclusion about radiation dose-response because of the consistent chemotherapy administration in this population.

## Conclusions

In summary, our study showed that patients with BED > 57 Gy had significantly better LC, PFS and OS than those with BED ≤ 57 Gy in LS-SCLC population treated with TRT physical dose ≥ 50 Gy, indicating that a biologically dose-response relationship still existed even in a relatively high radiation dose range for LS-SCLC. However, the data on toxicities for LS-SCLC treated with high BED is still limited, especially with modern irradiation technique. A prospective phase I/II study of accelerated three-dimensional conformal hypofractionated TRT with 55 Gy in 22 fractions over 30 days (BED 62 Gy) plus concurrent chemotherapy in patients with LS-SCLC is ongoing in our centre, with the hypothesis that both high TRT dose and short ORT are important for the treatment of LS-SCLC.

## Competing interests

The authors declare that they have no competing interests.

## Authors' contributions

BX and XLF designed this study, performed much of the work, and drafted the manuscript. Patient accrual and clinical data collection was done by all authors. XWC and JDZ participated in the analysis and the data interpretation. All authors read and approved the final manuscript.
